# Estudio de la estabilidad de la actividad lactato deshidrogenasa en plasma a distintas temperaturas: conservación post-analítica

**DOI:** 10.1515/almed-2020-0025

**Published:** 2020-05-19

**Authors:** Marta Gómez López, Neila Rodríguez Roca, Manuela Simón Velasco, María José Alcaide Martín, Antonio Buño Soto, Rubén Gómez Rioja

**Affiliations:** Servicio de Análisis Clínicos, Hospital Universitario La Paz, Madrid, España

**Keywords:** estabilidad, lactato deshidrogenasa (LDH), post-analítica

Estimado Editor,

Las condiciones de almacenamiento de las muestras son un factor clave en la pre-analítica y post-analítica del laboratorio clínico. Habitualmente, la conservación post-analítica de las muestras se realiza en refrigeración. El frío disminuye el metabolismo celular y reduce las reacciones de degradación de muchas magnitudes, pero puede no ser la condición de conservación óptima en todos los casos. Este parece ser el caso de la enzima lactato deshidrogenasa (LDH), aunque, en la bibliografía no existe un consenso claro acerca de la temperatura de conservación idónea. Por ello, decidimos realizar un estudio siguiendo el protocolo publicado por la Sociedad Española de Medicina de Laboratorio (SEQC^ML^) [1], para determinar el límite de estabilidad de LDH en muestras de plasma a temperatura ambiente (TA) o refrigerada.

Se realizó un estudio preliminar con determinación basal y a las 48 horas, que es el tiempo de retención post-analítico habitual en nuestro laboratorio. Se fijó la diferencia máxima admisible (DMA) en función de las especificaciones de calidad del laboratorio (6,39%, error sistemático mínimo calculado según la fórmula de VB en la Base de Datos de la página de la SEQC^ML^) [2].

Se utilizaron 3 muestras sobrantes de plasma heparina de litio y se hicieron 3 alícuotas de cada muestra; una alícuota basal, que se congeló a −80 °C inmediatamente (muestra basal) y dos alícuotas que se conservaron a TA y a 4−8 °C. Transcurrido el tiempo establecido, se conservaron todas las alícuotas a −80 °C. Posteriormente se descongelaron, se homogeneizaron y se analizaron en la misma serie. La actividad de LDH se determinó mediante método Lactatopiruvato según IFCC en el analizador Dimension Vista 1500 (Siemens Healthineers). Las determinaciones se efectuaron por sextuplicado considerando el cociente entre la DMA y la imprecisión analítica habitual en nuestro laboratorio (CV = 2,97%).

La pérdida de estabilidad se expresó mediante la diferencia porcentual (DP%) obtenida con la media de los replicados para cada muestra, según la ecuación DP% = T_tiempo_ − T_basal_/T_basal_ × 100. Se observa que a TA no hay una variación significativa (DP% < DMA). En cambio, las muestras refrigeradas tienen una pérdida en torno al 12–15%, superior a la DMA (Tabla 1).

**Tabla 1:** Resumen de resultados de estudio preliminar.

	Basal	T^a^ ambiente	Nevera
Paciente 1			
Media	591,83	611,50	518,00
SD	9,83	16,47	3,22
CV%	1,66	2,69	0,62
DP%		3,32	−12,48
DMA%		6,39%	6,39%
Paciente 2			
Media	242,67	248,33	211,67
SD	3,20	5,16	3,44
CV%	1,32	2,08	1,63
DP%		2,34	−12,77
DMA%		6,39%	6,39%
Paciente 3			
Media	259,17	251,83	219,00
SD	1,47	1,33	3,52
CV%	0,57	0,53	1,61
DP%		−2,83	−15,50
DMA%		6,39%	6,39%

DP, diferencia porcentual; DMA, diferencia máxima admisible.

Para definir el límite de estabilidad en refrigeración se efectuó un estudio extendido. Se utilizaron 10 muestras de plasma, de las que se hicieron siete alícuotas, correspondientes a cada uno de los tiempos estudiados: basal, 12, 24, 36, 48, 60 y 72 horas que se conservaron a 4–8 °C. Las alícuotas almacenadas en nevera se fueron congelando a −80 °C en cada tiempo de estudio. Transcurrido el tiempo establecido, todas las alícuotas se descongelaron, se homogeneizaron y se analizaron en la misma serie por duplicado.

Se define una ecuación de inestabilidad (DP% = −0,14 × tiempo(h)) ajustada por mínimos cuadrados, obteniendo un coeficiente de correlación de Pearson (R) de 0,87 y una pendiente significativamente distinta de cero. Según esta ecuación, hay una pérdida de estabilidad diaria del 3,4%. Es decir, para una DMA de 6,39% se obtiene un límite de estabilidad de 45 horas (Figura 1).

**Figura 1:**
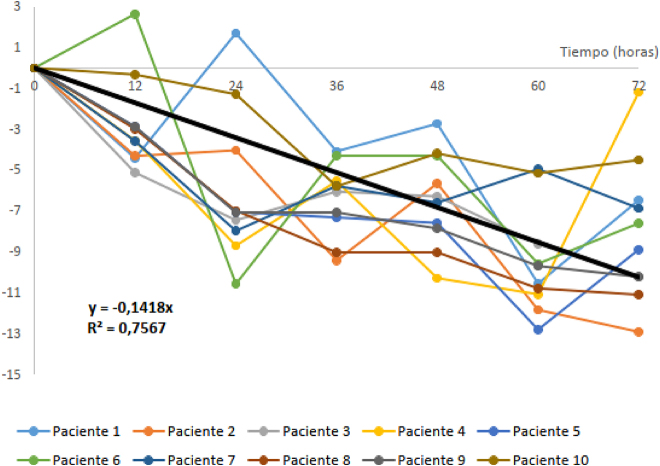
Representación gráfica del estudio extendido.

Nuestros resultados coinciden con lo que observan Shimizu Y, An B y Heins M, [3], [4], [5] que exponen que la actividad disminuye en muestras refrigeradas mientras que se mantiene estable a TA. Sin embargo, Oddoze C [6] observó un aumento de la actividad. Los límites de estabilidad mencionados oscilan entre 1 y 4 días, posiblemente en relación a los diferentes criterios de DMA que varían entre 3,53 y 7%.

La causa de la pérdida de estabilidad de la LDH en frío podría estar en la distinta termosensibilidad de las isoenzimas de la LDH. La LDH-4 y 5 son especialmente lábiles y posiblemente las responsables de la pérdida de actividad en frío [4]. Ésta podría ser también la explicación a la gran variabilidad interindividual observada y podría ser la causa de que los 3 pacientes incluidos en el estudio preliminar presentaran una pérdida de estabilidad ligeramente mayor a la predicha finalmente con el modelo extendido, que incluye un mayor número de pacientes.

La información sobre estabilidad de las magnitudes bioquímicas disponible es muchas veces confusa. La verificación de los límites de estabilidad en cada laboratorio permite asegurar la adecuación del límite de estabilidad a las condiciones particulares de cada laboratorio como el tipo de tubo, temperatura, luz o agitación. El estudio preliminar propuesto por la SEQC^ML^ permite confirmar la validez de un límite de estabilidad habitual, adaptado a la rutina del laboratorio, de forma simple y poco costosa. En caso de observarse posibles pérdidas de estabilidad la realización de un estudio extendido permite calcular una ecuación de inestabilidad que puede ser compartida con laboratorios que trabajen en condiciones similares.

## Supplementary Material

Supplementary Material Details

## Material Suplementario

La versión en línea de este artículo ofrece material suplementario (https://doi.org/10.1515/almed-2020-0025).
