# Supplementary material for: Estudio de la estabilidad de la actividad lactato deshidrogenasa en plasma a distintas temperaturas: conservación post-analítica
Source: Adv Lab Med. 2020 May 19;1(4):20200025. [Article in Spanish] doi: 10.1515/almed-2020-0025 (PMC10197260; doi:10.1515/almed-2020-0025)
Supplement: Supplementary file 1 — Supplementary Material Details [file j_almed-2020-0025_suppl.pdf]

ANEXOS

Anexo 1. Datos completos estudio preliminar en Dimension Vista 1500.

	Paciente 1		
	**Basal**	**Tª ambiente**	**Nevera**
**Repetición 1**	602	613	522
**Repetición 2**	601	627	519
**Repetición 3**	598	620	521
**Repetición 4**	588	599	515
**Repetición 5**	583	625	517
**Repetición 6**	579	585	514
**Media**	591,83	611,50	518,00
**SD**	9,83	16,47	3,22
**CV%**	1,66	2,69	0,62
**DP%**		3,32	-12,48
**DMA%**		6,39%	6,39%

	Paciente 2		
	**Basal**	**Tª ambiente**	**Nevera**
**Repetición 1**	248	257	217
**Repetición 2**	245	245	214
**Repetición 3**	240	251	211
**Repetición 4**	241	249	211
**Repetición 5**	240	245	210
**Repetición 6**	242	243	207
**Media**	242,67	248,33	211,67
**SD**	3,20	5,16	3,44
**CV%**	1,32	2,08	1,63
**DP%**		2,34	-12,77
**DMA%**		6,39%	6,39%

	Paciente 3		
	Basal	Tª ambiente	Nevera
**Repetición 1**	260	250	219
**Repetición 2**	261	252	223
**Repetición 3**	258	251	222
**Repetición 4**	260	252	218
**Repetición 5**	257	252	219
**Repetición 6**	259	254	213
**Media**	259,17	251,83	219,00
**SD**	1,47	1,33	3,52
**CV%**	0,57	0,53	1,61
**DP%**		-2,83	-15,50
**DMA%**		6,39%	6,39%

Anexo 2. Datos completos estudio extendido Dimension Vista 1500.

		Basal	12 horas	24 horas	36 horas	48 horas	60 horas	72 horas
**Paciente 1**	**Repetición 1**	147	142	151	139	141	133	136
	**Repetición 2**	147	139	148	143	145	130	139
	**Media**	147	140,5	149,5	141	143	131,5	137,5
	**SD**	0,00	2,12	2,12	2,83	2,83	2,12	2,12
	**CV%**	0,00	1,51	1,42	2,01	1,98	1,61	1,54
**Paciente 2**	**Repetición 1**	185	178	178	167	175	161	160
	**Repetición 2**	187	178	179	170	176	167	164
	**Media**	186	178	178,5	168,5	175,5	164	162
	**SD**	1,41	0,00	0,71	2,12	0,71	4,24	2,83
	**CV%**	0,76	0,00	0,40	1,26	0,40	2,59	1,75
**Paciente 3**	**Repetición 1**	213	205	195	203	201	195	193
	**Repetición 2**	217	203	203	201	202	198	193
	**Media**	215	204	199	202	201,5	196,5	193
	**SD**	2,83	1,41	5,66	1,41	0,71	2,12	0,00
	**CV%**	1,32	0,69	2,84	0,70	0,35	1,08	0,00
**Paciente 4**	**Repetición 1**	126	122	115	122	113	111	125
	**Repetición 2**	127	122	116	117	114	114	125
	**Media**	126,5	122	115,5	119,5	113,5	112,5	125
	**SD**	0,71	0,00	0,71	3,54	0,71	2,12	0,00
	**CV%**	0,56	0,00	0,61	2,96	0,62	1,89	0,00
**Paciente 5**	**Repetición 1**	191	189	179	178	179	168	177
	**Repetición 2**	192	183	177	177	175	166	172
	**Media**	191,5	186	178	177,5	177	167	174,5
	**SD**	0,71	4,24	1,41	0,71	2,83	1,41	3,54
	**CV%**	0,37	2,28	0,79	0,40	1,60	0,85	2,03
**Paciente 6**	**Repetición 1**	148	157	137	145	147	137	144
	**Repetición 2**	155	154	134	145	143	137	136
	**Media**	151,5	155,5	135,5	145	145	137	140

	**SD**	4,95	2,12	2,12	0,00	2,83	0,00	5,66
	**CV%**	3,27	1,36	1,57	0,00	1,95	0,00	4,04
**Paciente 7**	**Repetición 1**	178	174	171	173	168	170	169
	**Repetición 2**	186	177	164	170	172	176	170
	**Media**	182	175,5	167,5	171,5	170	173	169,5
	**SD**	5,66	2,12	4,95	2,12	2,83	4,24	0,71
	**CV%**	3,11	1,21	2,96	1,24	1,66	2,45	0,42
**Paciente 8**	**Repetición 1**	494	486	448	442	441	436	431
	**Repetición 2**	480	459	458	444	445	433	435
	**Media**	487	472,5	453	443	443	434,5	433
	**SD**	9,90	19,09	7,07	1,41	2,83	2,12	2,83
	**CV%**	2,03	4,04	1,56	0,32	0,64	0,49	0,65
**Paciente 9**	**Repetición 1**	191	183	179	176	178	171	172
	**Repetición 2**	191	188	176	179	174	174	171
	**Media**	191	185,5	177,5	177,5	176	172,5	171,5
	**SD**	0,00	3,54	2,12	2,12	2,83	2,12	0,71
	**CV%**	0,00	1,91	1,20	1,20	1,61	1,23	0,41
**Paciente 10**	**Repetición 1**	154	153	154	145	151	147	147
	**Repetición 2**	157	157	153	148	147	148	150
	**Media**	155,5	155	153,5	146,5	149	147,5	148,5
	**SD**	2,12	2,83	0,71	2,12	2,83	0,71	2,12
	**CV%**	1,36	1,82	0,46	1,45	1,90	0,48	1,43
